# Assessing the correlation between perceived stress and academic achievement among health sciences students

**DOI:** 10.3389/fmed.2025.1734838

**Published:** 2026-01-09

**Authors:** Muhammad Al-Roomy

**Affiliations:** King Abdullah International Medical Research Center (KAIMRC), King Saud bin Abdulaziz University for Health Sciences, Ministry of National Guard, Riyadh, Saudi Arabia

**Keywords:** academic achievement, anxiety, medical students, perceived stress, performance

## Abstract

**Introduction:**

University students are vulnerable to a myriad of academic challenges that can impede their learning processes and lead to adverse educational outcomes. Perceived stress among students is one such challenge. Objective: this study’s objective was to examine the influence of perceived stress on academic performance among health sciences students.

**Methods:**

A total of 210 first-year health sciences students in their second semester were recruited from different Saudi universities in the Riyadh region. An online questionnaire was administered to the health sciences cohort using the Perceived Stress Scale (PSS). This instrument comprises 14 items designed to assess emotions and thoughts experienced over the preceding month. The data obtained were analyzed using IBM SPSS Statistics (version 27.0) to compute descriptive statistics, including means, standard deviations, ranges, frequencies, and percentages, to summarize participants’ demographic characteristics, perceived stress levels, and measures of academic achievement.

**Results:**

The results indicated that a significant proportion of students experienced frequent stress at varying intensities, along with difficulties in managing it. The majority of the participants (74.8%) were categorized as having moderate stress, whereas 14.3% reported high stress and 11.0% reported low stress. Furthermore, the analysis revealed significant negative correlations between perceived stress and academic performance in health science courses, implying that students with elevated stress levels tended to attain lower academic scores. Ultimately, the findings suggested that the students experiencing heightened stress typically exhibited diminished academic performance, as reflected in their cumulative grade point average (GPA). These findings underscore the importance of addressing the issue of perceived stress among students, advocating for various courses of action for students, educators, and policymakers.

## Introduction

Stress has been defined in various ways by scientists, depending on their disciplinary perspectives in fields such as psychology, medicine, and sociology (1). According to Almadi (2), the literature contains three dominant approaches to defining stress. The first views stress as a stimulus or external event. The second regards it as a response or an internal mechanism, i.e., people respond to environmental stressors differently. The third defines stress as a dynamic interaction between individuals and their environment, focusing on how it is perceived and dealt with in this transactional phase.

Individuals’ ability to adjust to stressors is crucial for responding to the imbalance that stress causes (1). Gibbons (3) emphasized that students’ perception of stress can be divided into two facets: distress and eustress. The negative perception of stress that hampers progress is called distress. On the other hand, the positive perception of stress, which presents a challenge that can help individuals tap into their potential and make progress, is called eustress.

University students are susceptible to several study obstacles that can hinder their learning and result in negative academic outcomes, affecting their resilience and success. Stress is one of them (4–9). This is because the life of a university student is inherently demanding, marked by a complex interplay of academic, social, and personal challenges. Students experience certain stressors inside classrooms, such as the relentless demands of a rigorous curriculum and heavy academic workloads. Such perceived stress is considered a significant concern and leads to consequences including poor academic performance, several physical and mental health problems, poor overall wellbeing, and behavioral changes (10).

Regardless the sources of stressors, Ghatol (8) asserted that stress leads to depression, which is the most common health problem experienced by college students. He also highlighted the negative impact of stress on academic performance (8) (p. 2) argued that “stress makes a significant contribution to the guess/prediction of subsequent student behavior and performance and thus act as a negative predictor of academic performance of student” [sic]. Krashen (11) observed that reducing students’ anxiety and classroom stressors allows them to work at their own pace and perform better. This can be achieved by elevating coping skills to control stress by fostering resilience and motivation (12–14).

Based on a review of the literature, few studies have been conducted in the Saudi context. More specifically, no study has examined the correlation between perceived stress and academic achievement among first-year students. Therefore, this study aimed to fill this gap and explore the impact of perceived stress on students’ academic performance.

### Studies on perceived stress among students

Previous research has addressed the issue of perceived stress and its impact on students’ academic performance across different contexts.

For example, Cao et al. (15) recently examined the relationships between perceived stress, emotions, and academic procrastination among higher vocational nursing students. The study revealed that the majority of nursing students (more than 80%) reported academic procrastination, and there was a significant positive correlation between perceived stress, negative emotions, and academic procrastination. Moreover, the study also found that both negative and positive emotions were partial mediators between perceived stress and academic procrastination.

In addition, a cross-sectional study by Ebrahim et al. (16) explored the impact of perceived stress and anxiety on medical students’ academic performance. The findings reported that approximately 93 medical students experienced moderate-to-high stress levels, while more than half experienced moderate-to-concerning levels of anxiety. The main stressors found among students were related to academics and teaching. Using a different sample, Seedhom et al. (17) conducted a cross-sectional study to distinguish between medical and non-medical students’ stress levels and their predictors. They found that stress levels were slightly higher among medical students than among non-medical students, while both groups reported academic stressors as significant predictors of perceived stress. In a different context involving international students, Ali et al. (18) investigated the correlation between cumulative stress and certain stressors and found that overall language proficiency, academic adjustments, and adjustment to college life and living were the stressors most frequently reported by students.

In the Saudi context, Rahman et al. (19) found that medical students reported high stress levels. The most frequently occurring stressors among students were related to academic and psychosocial domains. Another study by El-Zayat et al. (20) revealed a significant correlation between emotional eating and perceived stress among Saudi university students. This study also found that academic stress, pressure to succeed, family pressure, and financial factors were the stressors most commonly encountered by students. Meanwhile, Albasheer et al. (21) conducted a cross-sectional study to investigate the prevalence of depression, anxiety, and stress symptoms, as well as barriers to accessing mental health services, among medical students. The results indicated that 49.2% of students were normal, while 11.0, 16.7, 14.1, and 9.0% experienced mild, moderate, severe, and extremely severe levels of stress, respectively.

Similarly, Al-Garni et al. (22) performed a study to examine the prevalence of depression, anxiety, and stress among students in health and non-health science courses. Their findings indicated that 54.2% of students showed normal levels of stress, while 33.5% experienced mild stress. Furthermore, stress levels were higher among health sciences students than among non-health sciences students (44.58 vs. 47.44). Aljaffer et al. (23) considered the impact of coping strategies and explored the connection between coping mechanisms, stress, and anxiety among medical students. Their findings revealed that the majority of participants experienced moderate-to-high stress levels (72%). Moreover, stress levels exhibited a moderate positive association with coping strategies.

Finally, Shubayr and Dailah (24) explored the relationships between emotional intelligence, self-efficacy, and perceived stress. The study found a positive correlation between the emotional intelligence domain of self-efficacy and perceived stress and a negative correlation between emotional intelligence and perceived stress.

Based on the abovementioned research and the identified research gap, three research questions were formulated for this investigation:

1. What is the stress level of health sciences students in the preparatory year?
2. Is there a significant correlation between students’ stress levels and their academic performance in science courses, specifically Biology, Chemistry, and Physics?
3. Is there a significant correlation between students’ stress levels and their overall grade point average (GPA)?

## Methods

### Sample

A total of 210 first-year health sciences students, aged 18–20 years, were selected during their second semester from three Saudi universities in the Riyadh region. A convenience sampling technique, i.e., a non-probability sampling method, was employed to collect data from the participants. This method was adopted due to its efficiency in conserving resources, time, and effort, as it has been proven challenging for the researcher to visit multiple classes to reach the students.

All participants lived in Riyadh. Prior to data collection, informed consent was obtained from the participants, outlining the study’s objectives and informing them of their right to withdraw at any time or not participate without any consequences. An online questionnaire using the Perceived Stress Scale (PSS) was made available for 2 weeks to approximately 350 students, of whom 210 willingly completed it.

### Research instrument

The midterm exam grades in three science courses—Chemistry, Biology, and Physics—were gathered, along with the overall cumulative GPA on a 5-point scale, and correlated with the students’ perception of stress. The science scores were standardized across sections, all following the 5-point grading scale.

In addition, an online questionnaire (i.e., the PSS) was administered to health sciences students in the preparatory year. The students had to complete the questionnaire anonymously within 15–20 min. The questionnaire was developed by Cohen et al. (25). It is “a 14-item measure of the degree to which situations in one’s life are appraised as stressful.” It is more comprehensive than previous life-event scales due to its sensitivity to ongoing stress events. Specifically, it captures chronic stress resulting from persistent life situations, stress from unlisted occurrences, and reactions to specific events included in any scale (25).

In this questionnaire, the 14 items assess students’ feelings and thoughts over the past month. Seven items (4–7, 9, 10, and 13) are positively stated, while the remaining seven items are negatively stated. The students responded to the items using a 5-point Likert scale ranging from 0 (“never”) to 4 (“very often). For the seven positive items (4–7, 9, 10, and 13), the 5 Likert scale is reversed, so 0 represents “very often” and 4 represents “never.” The total score ranges from 0 to 56 and is divided into three levels: low stress (0–18), moderate stress (19–37), and high stress (38–56). The items ask direct questions about the level of stress experienced daily over the past month; it is more likely that the predictive validity of the assessed stress will change significantly after 4–8 months. Although the PSS was originally developed to assess junior high school students (25), it has since been widely employed as a psychological measure of perceived stress (26).

To ensure the clarity and comprehensibility of the items, as well as to evaluate their internal validity, the questionnaire was piloted with three English educators and 15 students, and their observations and feedback were duly considered. In addition, a reliability analysis was conducted on the 14 items of the PSS. Cronbach’s alpha showed that the scale demonstrated acceptable reliability (*α* = 0.74).

### Statistical analysis

All statistical analyses were conducted using IBM SPSS Statistics version 27.0 (IBM Corp., Armonk, NY, United States). Descriptive statistics, including means, standard deviations, ranges, frequencies, and percentages, were calculated to summarize the participants’ demographic characteristics, perceived stress scores, and academic achievement measures (GPA and science course scores in Physics, Biology, and Chemistry). Total PSS scores were computed and categorized into low, moderate, and high stress levels based on the established cut-off values.

To address the research questions, inferential statistical analyses were performed. Pearson’s correlation coefficients were used to examine the relationships between perceived stress and academic achievement (GPA and science course scores). Where appropriate, Spearman’s rho was considered for non-normally distributed variables.

To investigate the differences in academic performance across the three stress categories, the assumptions of normality and homogeneity of variance were first assessed using the Shapiro–Wilk test and Levene’s test, respectively. Due to the violation of the assumption of homogeneity of variance across the groups, Welch’s one-way analysis of variance (ANOVA) was conducted for all academic variables (GPA, Physics, Biology, and Chemistry) instead of the standard *F*-test. Significant main effects were followed by Games–Howell post-hoc pairwise comparisons, which are robust to unequal variances and sample sizes. Effect sizes were calculated using eta squared (*η*^2^) to evaluate the magnitude of group differences and were interpreted according to Cohen’s conventional benchmarks (small = 0.01, medium = 0.06, and large = 0.14). A two-tailed significance level of *p* < 0.05 was considered statistically significant for all analyses.

## Results

### Perceived Stress Scale scores

To address the first research question, “What is the stress level of health sciences students?,” the students’ stress levels were assessed. The participants’ responses to the 14 PSS items revealed a diverse pattern of perceived stress experiences (Supplementary Appendix 1). A considerable proportion of students reported frequent feelings of stress and difficulty coping. For instance, almost one-third of the students (30.0%) reported feeling nervous and stressed “fairly often,” while an additional 21.4% experienced these feelings “very often.” Similarly, 27.1% of the students stated that difficulties were accumulating “fairly often,” and 21.4% reported this occurring “very often.” These results suggest that many students perceived themselves as being under persistent strain.

On the other hand, responses to positive coping items (reverse-coded items) showed that many students retained some confidence in their ability to manage. For example, 35.2% of the students reported “sometimes” feeling confident about handling personal problems, and 30.0% reported this occurring “fairly often.” Similarly, 43.8% of the students indicated that they were “sometimes” able to control irritations, and 29.5% reported this occurring “fairly often.” These results suggest that although stress levels were high, the students perceived themselves as having at least a moderate ability to manage daily challenges.

**Figure 1:**
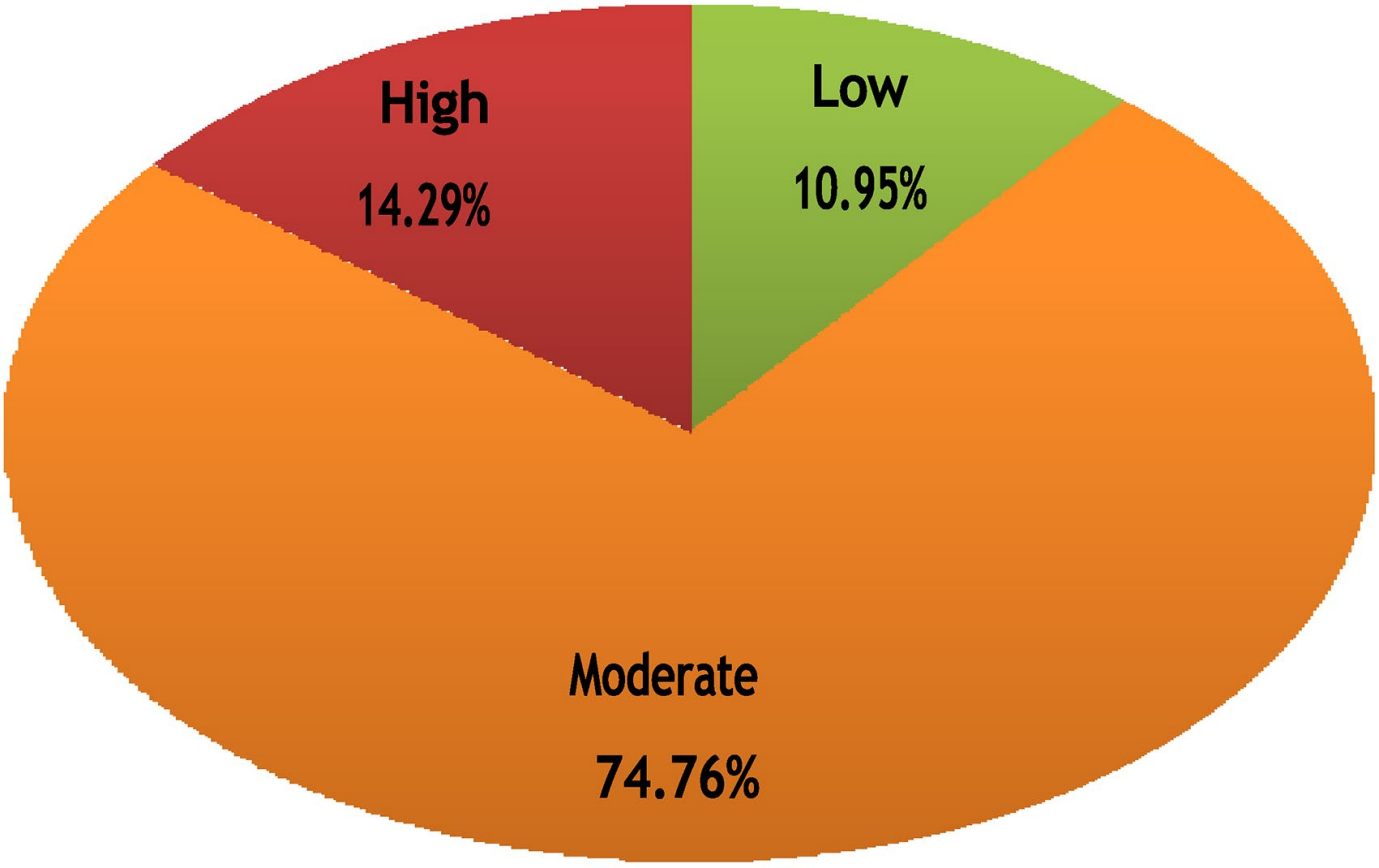
Distribution of the health sciences students according to the stress level.

The total PSS scores aligned with the item-level findings, indicating that the students generally experienced moderate stress levels. The mean score was 28.70 (SD = 8.45; range = 4–56). Based on established cutoff values, the majority of the participants (74.8%) fell within the moderate stress category, while 14.3% reported high stress and 11.0% reported low stress (Figure 1). These results emphasize that stress was prevalent among most health sciences students, with a notable subset at risk for elevated stress levels.

### Students’ academic performance

To address the second research question, “Is there a significant correlation between students’ stress levels and their academic performance in science courses, specifically Biology, Chemistry, and Physics?,” the students’ academic performance was assessed using their overall grade point average (GPA) and scores in the three science courses: Physics, Biology, and Chemistry. Descriptive statistics are presented in Table 1. The mean GPA was 4.42 (SD = 0.58), with scores ranging from 3.00 to 5.00. The distribution suggests that most students maintained a relatively high academic standing, as reflected by a median GPA of 4.62.

**Table 1:** Descriptive statistics for GPA and science course scores.

Variables	Min.	Max.	Mean	Median	SD
Physics	10.0	25.0	21.5	23.0	3.6
Biology	8.0	35.0	27.2	29.0	6.3
Chemistry	6.0	60.0	43.6	47.0	12.6
GPA	3.0	5.0	4.4	4.6	0.6

For individual science courses, the mean score was 21.50 (SD = 3.58; range = 10–25) for Physics, 27.24 (SD = 6.31; range = 8–35) for Biology, and 43.63 (SD = 12.58; range = 6–60) for Chemistry. Median scores were highest in Chemistry (47.00), followed by Biology (29.00) and Physics (23.00). These results indicate that, overall, the students performed better in Chemistry compared to Biology and Physics. Collectively, the findings show that the students demonstrated above-average achievement across GPAs and science courses, with some variability across disciplines.

### Relationship between stress and academic achievement

Regarding the third research question, “Is there a significant correlation between students’ stress levels and their overall GPAs?,” the results showed a moderate-to-strong negative correlation between perceived stress and the overall GPA.

Pearson correlation analyses revealed significant negative associations between perceived stress and performance in the science courses (Table 2). Higher PSS scores were associated with lower achievement in Physics (*r* = −0.23, *p* = 0.001), Biology (*r* = −0.34, *p* < 0.001), and Chemistry (*r* = −0.34, *p* < 0.001). Among the three science subjects, the strongest negative correlation was observed in Biology and Chemistry, suggesting that the students with higher stress levels tended to achieve lower scores in these courses.

**Table 2:** Correlations between perceived stress (PSS) and academic achievement.

Variables	PSS score	GPA	Physics	Biology	Chemistry
PSS Score	–				
GPA	−0.57*	–			
Physics	−0.23*	0.55*	–		
Biology	−0.34*	0.55*	0.47*	–	
Chemistry	−0.34*	0.63*	0.44*	0.52*	–

*Significant correlation at the 0.001 level.

Similarly, there was a significant moderate-to-strong negative correlation between perceived stress and the overall GPA (*r* = −0.57, *p* < 0.001). This finding indicates that the students who experienced higher stress levels generally demonstrated lower academic performance, as reflected in their cumulative GPA.

### Impact of stress levels on academic performance

To examine the impact of stress levels on academic performance, Welch’s one-way ANOVA was conducted to compare the overall GPA and science course scores (Physics, Biology, and Chemistry) across the three stress categories (low, moderate, and high). Welch’s *F*-test was selected because the assumption of homogeneity of variance was violated, as indicated by a significant Levene’s test (*p* < 0.05), and Welch’s *F*-test does not assume equal variances and is robust to unequal sample sizes (27). The assumption of normality was checked using the Shapiro–Wilk test. Although the data deviated from a normal distribution, the sample size was sufficiently large (*N* = 210, *n* > 3 per group) to assume the reliability and robustness of the *F*-test results (28).

The analyses revealed statistically significant differences in all academic outcomes across the stress levels (Table 3).

**Table 3:** One-way ANOVA results for academic achievement by stress level.

Variable	Stress level	Mean	SD	*F*-value	*p*-value	*η*^2^ (95% CI)
GPA	Low	4.9^a^	0.2	54.45	<0.001	0.224 (0.128–0.312)
	Moderate	4.5^b^	0.5			
	High	3.8^c^	0.6			
Physics	Low	22.8^a^	2.2	6.00	<0.01	0.07 (0.014–0.139)
	Moderate	21.7^a^	3.3			
	High	19.3^b^	4.9			
Biology	Low	29.9^a^	5.2	9.34	<0.001	0.102 (0.033–0.179)
	Moderate	27.7^a^	5.9			
	High	22.6^b^	7.2			
Chemistry	Low	50.3^a^	8.8	9.46	<0.001	0.065 (0.012–0.133)
	Moderate	43.8^b^	12.5			
	High	37.5^c^	12.8			

Superscripts (^a^, ^b^, ^c^) indicate significant differences between the groups based on Games–Howell *post-hoc* comparisons (*p* < 0.05). η^2^, Eta squared.

For GPA, there was a large and significant effect of stress level [Welch’s *F*(2, 61.84) = 54.45, *p* < 0.001, *η*^2^ = 0.22]. Games–Howell post-hoc comparisons indicated that the students with low stress (Mean = 4.9, SD = 0.2) achieved significantly higher GPAs than those with moderate stress (Mean = 4.5, SD = 0.5), while students with high stress (Mean = 3.8, SD = 0.6) scored significantly lower than both other groups.

For Physics, a significant effect was also found [Welch’s *F*(2, 45.11) = 6.00, *p* < 0.01, *η*^2^ = 0.07]. The post-hoc comparison showed no significant differences between the students with low stress (Mean = 22.8, SD = 2.2) and those with moderate stress (Mean = 21.7, SD = 3.3). Nonetheless, both groups scored significantly higher than the students with high stress (Mean = 19.3, SD = 4.9).

For Biology, Welch’s ANOVA indicated a significant difference across the groups [Welch’s *F*(2, 42.92) = 9.34, *p* < 0.001, *η*^2^ = 0.10]. Similar to the Physics results, Games–Howell comparisons showed no significant difference between the students with low stress (Mean = 29.9, SD = 5.2) and those with moderate stress (Mean = 27.7, SD = 5.9). However, both groups significantly outperformed their highly stressed peers (Mean = 22.6, SD = 7.2).

Similarly, Chemistry performance differed significantly across stress levels [Welch’s *F*(2, 47.45) = 9.46, *p* < 0.001, *η*^2^ = 0.07]. The students with low stress achieved the highest scores (Mean = 50.3, SD = 8.8), followed by those with moderate stress (Mean = 43.8, SD = 12.5), while the highly stressed students performed the lowest (Mean = 37.5, SD = 12.8). Across all outcomes, the effect sizes (*η*^2^ = 0.07–0.224) indicated moderate-to-large effects, suggesting that stress levels had a meaningful impact on the students’ academic achievement.

## Discussion

The above findings are consistent with previous studies reporting that health sciences students experience moderate-to-high stress levels (19, 24). They also align with studies showing that medical students tend to experience higher stress levels compared to non-medical students (17). However, in this study, the mean PSS score was 28.70, indicating a moderate perceived stress level. This is lower than that reported in studies conducted in different contexts (Egypt, China), where mean scores were 31.76 ± 8.45, 50.36 ± 12.58, and 97.4 ± 21.83 (15, 16, 18).

This study found that 74.79% of the students reported moderate stress levels, 14.29% reported high stress levels, and 10.95% reported low stress levels in the same environment. This clearly highlights how students respond differently to different stressors.

However, the students were able to develop coping strategies to manage stressors such as challenging situations and negative emotions. Their responses to positive items (4–7, 9, 10, and 13) were mostly “sometimes” and “fairly often.” These replies suggest that, although stress levels were high, the students perceived at least a moderate ability to manage daily challenges. By responding to the imbalance caused by stress, the students were able to adjust stressors effectively and adapt to environmental threats (1). Some students might see academic demands as something good that motivates them to challenge themselves and reach their potential. Others interpret such situations as harmful or as threats that hinder their progress. The dynamic interplay between students’ perceptions of stressors and their environment, and how they cope with and overcome stressors, determines their stress response (1).

This finding is similar to that of Sani et al. (29) study conducted in the Saudi context, which found that students’ stress levels decreased as they progressed through their years of study. Another study found a similar result, where 72 students reported moderate stress levels (23).

One explanation could be that this study’s participants had the advantage of not having risk factors that could lead to higher perceived stress, such as being female, living away from family, age and year of study, or being international students. These factors have been identified as significant stressors that can affect coping strategies in previous studies (30).

Interestingly, in some studies conducted in the Saudi context, mean PSS scores were much lower. For example, the mean score reported by Shubayr and Dailah (24) was 7.15 ± 2.11; similarly, in two other studies, students demonstrated much lower stress levels, with mean scores of 12.3 and 16.7 (21, 22). This could be attributed to other independent variables influencing perceived stress in those studies, such as participants’ age, gender, level of study, and field of study. For example, in Rahman et al.’s (19) study, the mean score was 22.79 ± 5.80 among male and female students in their third year or higher who were engaged in clinical practice. Meanwhile, the mean score in El-Zayat et al. (20) study was 26 ± 5. In this study, there was no statistically significant correlation between perceived stress and general characteristics, such as city, education, academic year of study, or most recent GPA, except for having a chronic illness.

One plausible explanation for this result is that, in the current study, the first-year students were placed in a competitive environment that subjected them to a filtering mechanism during the first year. While they were studying, they were required to compete for class rankings. By the end of the year, they were assigned to different colleges based on their performance in different courses and the overall GPA. For example, the highest-achieving students were placed into colleges of medicine, dentistry, pharmacy, public health and health informatics, and applied medical sciences.

Regarding the correlation between perceived stress and academic achievement, the current study found significant negative associations between perceived stress and performance in health science courses, suggesting that the students with higher stress levels tended to achieve lower scores in these courses. This finding is supported by Item 12 of the PSS questionnaire, “In the last month, how often have you found yourself thinking about things that you have to accomplish?” Cognitive and emotional stressors appeared particularly prominent. More than half of the participants (53.3%) reported that they “very often” found themselves thinking about things they had to accomplish, reflecting a strong tendency toward overthinking and mental preoccupation with academic tasks. In contrast, only a small percentage of students reported rarely thinking about tasks to be accomplished (6.2%).

Feelings of a lack of control also emerged as important. In their response to PSS Item 13, “In the last month, how often have you been able to control the way you spend your time?,” nearly one-third of the students (32.9%) reported “sometimes” being unable to control important things in life, and 24.3% indicated that this occurred “fairly often.” Similarly, 35.2% of the students reported being angered by uncontrollable events “sometimes,” while 21.4% reported that this occurred “very often.” These findings highlight that external, uncontrollable circumstances were perceived as frequent stressors. Students have small projects to work on outside of classrooms, individually or in groups. They have time limits by which they must finish, and doctors, who teach them, conduct ongoing assessments to gauge what they have done. Another concern is that due to the large size of the curriculum, students have to study the uncovered class materials at home. As a result, students struggle to overcome the challenges they face and tend to carry stress from college into their home environments. The students showed their inability to manage the large amount of required work in responses to PSS Item 14, “In the last month, how often have you felt difficulties were piling up so high that you could not overcome them?” Approximately 26 students responded “sometimes,” and 27 responded “fairly often” to this item.

Another finding was that the students experiencing higher stress levels generally demonstrated lower academic performance, as reflected in their cumulative GPA. This finding aligns with other studies (16, 17, 19, 20). Such a result is expected, as university students are susceptible to several academic challenges both inside and outside the classroom. In this study, all participants were first-year students in a transitional phase, having just graduated from high school. Stress is considered a risk factor for students’ behavior and achievements. Students who experience perceived academic-related stress are more likely to perform poorly (31).

Failing to control the consequences of unmanaged stress can lead to poor performance. This is even more intense and challenging for medical students, who face higher academic pressure and other burdens. Medical students experience stress even before they enroll in universities. The stress they encounter can lead not only to poor performance but also to poor mental health, decreased motivation, and an increased risk of dropout (7, 10). For this reason, academic achievement is a prime concern for all medical students.

Bin Abdulrahman et al. (5) emphasized the importance of identifying the characteristics of stressed medical students and providing a comprehensive support system to foster resilience and improve academic performance. This system should consider the nature of medical education, addressing both academic and personal aspects to promote a healthy balance (32).

### Limitations

The sample included 210 health sciences students, and the study was conducted at three Saudi universities among first-year health sciences students, which limited external validity. With respect to the questionnaire, the use of self-report measures introduced the possibility of response bias. In addition, using a cross-sectional design prevented causal inference.

## Conclusion and recommendation

This study aimed to explore the impact of perceived stress on academic achievement among health sciences students. The findings provide valuable insights into how perceived stress affects students’ academic achievement. Students reported moderate-to-high stress levels, with those experiencing higher stress tending to achieve lower scores in science courses and lower cumulative GPAs.

These findings suggest several courses of action for students, teachers, policymakers, and researchers, and they support the notion that perceived stress plays a role in students’ learning. One important implication of this study is for institutional policy: institutions can incorporate evidence-based interventions to address students’ stress levels. Such interventions can provide techniques to overcome stress, such as a wellbeing center, structured academic advising, time management and mindfulness training, a peer mentoring program, and support groups. Second, teachers should be aware of the interconnections between academic and non-academic stressors, both inside and outside the college environment, and act accordingly. Although stress is seen as part of students’ lives, teachers should know that it is temporary and not a cause of long-term mental health problems. Nonetheless, some stressors arise from the large volume of assignments, quizzes, and learning materials. By reducing these demands, teachers could mitigate academic stress among health sciences students. Finally, it would be valuable to conduct further research using a longitudinal design to investigate perceived stress among students at different stages within the Saudi context. Future studies should include larger and more varied samples, including both male and female students from other health science institutions and different university levels, and should consider additional independent variables, such as age and field of study. In addition, future studies may employ other data collection methods, such as interviews and self-reported journals.

## Data Availability

The original contributions presented in the study are included in the article/Supplementary material, further inquiries can be directed to the corresponding author.
