# Supplementary material for: Assessing the correlation between perceived stress and academic achievement among health sciences students
Source: Front Med (Lausanne). 2026 Jan 9;12:1734838. doi: 10.3389/fmed.2025.1734838 (PMC12827518; doi:10.3389/fmed.2025.1734838)
Supplement: Supplementary file 1 [file Table_1.pdf]

Appendix 1

Item Responses to the Perceived Stress Scale (PSS) Among Health Science Students

Question	Never	Almost Never	Sometimes	Fairly Often	Very Often
1. In the last month, how often have you been upset because of something that happened unexpectedly?	24 (11.4%)	29 (13.8%)	85 (40.5%)	48 (22.9%)	24 (11.4%)
2. In the last month, how often have you felt that you were unable to control the important things in your life?	23 (11%)	50 (23.8%)	69 (32.9%)	51 (24.3%)	17 (8.1%)
3. In the last month, how often have you felt nervous and "stressed"?	15 (7.1%)	27 (12.9%)	60 (28.6%)	63 (30%)	45 (21.4%)
4. In the last month, how often have you dealt successfully with day-to-day problems and annoyances?	7 (3.3%)	20 (9.5%)	82 (39%)	69 (32.9%)	32 (15.2%)
5. In the last month, how often have you felt that you were effectively coping with important changes that were occurring in your life?	9 (4.3%)	16 (7.6%)	79 (37.6%)	70 (33.3%)	36 (17.1%)
6. In the last month, how often have you felt	12 (5.7%)	25 (11.9%)	74 (35.2%)	63 (30%)	36 (17.1%)

Question	Never	Almost Never	Sometimes	Fairly Often	Very Often
confident about your ability to handle your personal problems?					
7. In the last month, how often have you felt that things were going your way?	17 (8.1%)	36 (17.1%)	86 (41%)	48 (22.9%)	23 (11%)
8. In the last month, how often have you found that you could not cope with all the things that you had to do?	15 (7.1%)	36 (17.1%)	70 (33.3%)	57 (27.1%)	32 (15.2%)
9. In the last month, how often have you been able to control irritations in your life?	8 (3.8%)	28 (13.3%)	92 (43.8%)	62 (29.5%)	20 (9.5%)
10. In the last month, how often have you felt that you were on top of things?	12 (5.7%)	32 (15.2%)	86 (41%)	56 (26.7%)	24 (11.4%)
11. In the last month, how often have you been angered because of things that happened that were outside of your control?	20 (9.5%)	28 (13.3%)	74 (35.2%)	43 (20.5%)	45 (21.4%)
12. In the last month, how often have you found yourself thinking about	4 (1.9%)	9 (4.3%)	28 (13.3%)	57 (27.1%)	112 (53.3%)

Question	Never	Almost Never	Sometimes	Fairly Often	Very Often
things that you have to accomplish?					
13. In the last month, how often have you been able to control the way you spend your time?	15 (7.1%)	47 (22.4%)	92 (43.8%)	40 (19%)	16 (7.6%)
14. In the last month, how often have you felt difficulties were piling up so high that you could not overcome them?	17 (8.1%)	37 (17.6%)	54 (25.7%)	57 (27.1%)	45 (21.4%)
